# Opioid Nonadherence Risk Prediction of Patients with Cancer-Related Pain Based on Five Machine Learning Algorithms

**DOI:** 10.1155/2024/7347876

**Published:** 2024-06-06

**Authors:** Jinmei Liu, Juan Luo, Xu Chen, Jiyi Xie, Cong Wang, Hanxiang Wang, Qi Yuan, Shijun Li, Yu Zhang, Jianli Hu, Chen Shi

**Affiliations:** ^1^Department of Pharmacy, Union Hospital, Tongji Medical College, Huazhong University of Science & Technology (HUST), Wuhan, China; ^2^Hubei Province Clinical Research Center for Precision Medicine for Critical Illness, Wuhan 430022, China; ^3^Cancer Center, Union Hospital, Tongji Medical College, Huazhong University of Science and Technology, Wuhan 430022, China

## Abstract

**Objectives:**

Opioid nonadherence represents a significant barrier to cancer pain treatment efficacy. However, there is currently no effective prediction method for opioid adherence in patients with cancer pain. We aimed to develop and validate a machine learning (ML) model and evaluate its feasibility to predict opioid nonadherence in patients with cancer pain.

**Methods:**

This was a secondary analysis from a cross-sectional study that included 1195 patients from March 1, 2018, to October 31, 2019. Five ML algorithms, such as logistic regression (LR), random forest, eXtreme Gradient Boosting, multilayer perceptron, and support vector machine, were used to predict opioid nonadherence in patients with cancer pain using 43 demographic and clinical factors as predictors. The predictive effects of the models were compared by the area under the receiver operating characteristic curve (AUC_ROC), accuracy, precision, sensitivity, specificity, and F1 scores. The value of the best model for clinical application was assessed using decision curve analysis (DCA).

**Results:**

The best model obtained in this study, the LR model, had an AUC_ROC of 0.82, accuracy of 0.82, and specificity of 0.71. The DCA showed that clinical interventions for patients at high risk of opioid nonadherence based on the LR model can benefit patients. The strongest predictors for adherence were, in order of importance, beliefs about medicines questionnaire (BMQ)-harm, time since the start of opioid, and BMQ-necessity. *Discussion*. ML algorithms can be used as an effective means of predicting adherence to opioids in patients with cancer pain, which allows for proactive clinical intervention to optimize cancer pain management. This trial is registered with ChiCTR2000033576.

## 1. Introduction

Cancer pain is an important and distressing symptom that tends to increase in frequency and intensity as cancer progresses [1]. More importantly, pain is an independent predictor of survival in cancer patients [2]. Thus, adequate pain management is critical for improving the quality of life and health outcomes in cancer patients.

There is a growing recognition that despite the many clinical guidelines that have been developed to help achieve effective cancer pain management, a large number of patients still have inadequate pain management. A systematic review reported approximately one-third of cancer patients still do not achieve adequate pain relief [3]. The reasons for inadequate pain management in cancer patients are multifaceted, and these barriers exist in all parts of the healthcare system, including healthcare professionals, patients, and healthcare system-related barriers [4]. Among the patient-related factors, one of the greatest barriers is poor adherence to analgesics [5]. Analgesics are the cornerstone of cancer pain management [6], and adherence to prescribed analgesic regimens is key to successful cancer pain management.

Adherence to analgesics in cancer pain patients has ranged from 8.9% to 82.0% in different studies due to the different methods to measure adherence [7]. Nonadherence is a multifactorial issue that may be influenced by a range of patient, disease, condition, social/economic, and healthcare system/team-related factors [7]. Therefore, identifying the various factors associated with pain medication adherence and timely interventions may help improve analgesic efficacy and achieve a better quality-of-life outcome in patients with cancer pain. Interventions to improve medication adherence have been intensively studied for decades, but even complex interventions have shown only modest, and sometimes ineffective, effects [7–10]. One possible explanation is that, as mentioned above, nonadherence is multifactorial and complete coverage of interventions is difficult to achieve [11]. Therefore, the field of adherence research has turned to new strategies that take an individualized rather than a standardized approach to adherence interventions [12, 13]. Another problem with the ineffectiveness of adherence interventions is that although a variety of factors are known to influence medication adherence, these factors vary across studies depending on their trial design and region, resulting in little knowledge of predictors of patients' actual analgesic-taking behavior [14, 15]. Consequently, constructing a patient medication adherence prediction model that allows clinical staff to anticipate in advance how patients will adhere to their pain medication is crucial, if possible, thus allowing them to adopt effective individualized interventions to improve patient adherence and thus analgesic efficacy.

In recent years, artificial intelligence methods such as machine learning (ML) have been increasingly used in medicine to predict clinical events [16]. ML is particularly well suited for analyzing large data sets, computing complex interactions, identifying hidden patterns, and generating actionable predictions in clinical settings. In many cases, ML has been shown to outperform traditional statistical techniques [17–20]. ML models offer a promising approach for predicting patient adherence to medications, and studies have been conducted in this area in diseases such as diabetes and hypertension with high prediction accuracy [14, 21–24]. However, to our knowledge, there have been no studies using ML methods to predict adherence to opioids the core component of moderate-to-severe cancer pain management in patients with cancer pain [6].

The study aims to investigate whether an ML approach can accurately predict nonadherence to opioids in patients with cancer-related pain based on a previous cross-sectional study. This approach allows (1) simultaneous analysis of dozens and hundreds of clinical variables; (2) automated prediction without additional screening steps, thus reducing the burden on healthcare professionals; and (3) the developed models could be implemented in a clinical setting in the form of a clinical decision support system (CDSS), which in turn could help prescribers design targeted interventions to improve patient outcomes.

## 2. Methods

### 2.1. Design and Study Population

**Figure 1:**
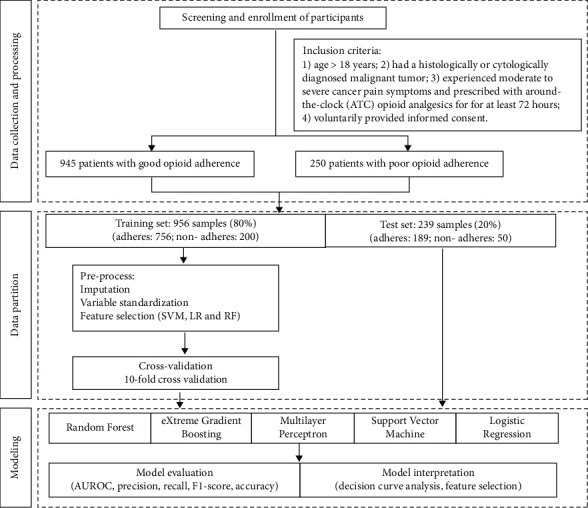
Flow diagram of the study.

Figure 1 shows the study process. This study is a secondary analysis of existing data from a parent study as an exploratory cross-sectional study of genetic and clinical factors associated with opioid response in Chinese Han patients with cancer pain [25]. Subjects were enrolled from March 2018 to October 2019 at a cancer center in a Grade III Level A hospital in central China. Patients were enrolled according to the following criteria: (1) age >18 years; (2) had a histologically or cytologically diagnosed malignant tumor; (3) experienced moderate-to-severe cancer pain symptoms and prescribed with around-the-clock (ATC) opioid analgesics for at least 72 hours; and (4) voluntarily provided informed consent. This study protocol was approved by the Medical Ethics Committee of Tongji Medical College, Huazhong University of Science and Technology (approval number: 2018-S016), and registration was submitted to the China Clinical Trials Registry (registration number: ChiCTR2000033576).

### 2.2. Outcome and Initial Predictor Selection

The outcome variable was dichotomous: adherence was defined as good if he/she is in strict accordance with the prescribed dose, frequency, and time of taking opioids at least 80% of the time; otherwise, it was defined as poor. Analgesic prescriptions were collected from medical records and how patients took them was reported by their self-reports. Nonadherence includes taking a lower/higher dose of opioids, self-discontinuation, forgetting to take medications, taking medications early/delayed, and taking other medications that are not on the prescription [26]. A total of 43 demographic and clinical-related factors, such as age, gender, diagnosis, Charlson comorbidity index (CCI), pain control, analgesic drugs used, and beliefs of medicine, were included as potential predictors. See Tables 1 and 2 for further details on the predictors. The measures of included variables had been well-explained elsewhere [25]. All categorical variables were transformed using a one-hot encoder before being used to construct the predictive model.

### 2.3. Missing Data Imputation

Seven of the 43 predictors had missing data, in the order of proportion missing: beliefs about medicines questionnaire (BMQ)-harm (13.39%), BMQ-overuse (13.39%), BMQ-necessity (11.72%), BMQ-concerns (11.72%), time since onset of pain symptoms (1.76%), time of tumor diagnosis (0.42%), and SDS score (0.17%) (Table 2). The missing data were relatively few and were not considered missing completely at random (MCAR); instead, their missingness was assumed to be related to the other measured variables (MAR). We estimate the values of missing data using k-nearest neighbor (k-NN) imputation, which is designed to help estimate missing data by finding the nearest neighbors of observations with missing data using the Euclidean distance metric and estimating the missing data based on the nonmissing values in the neighbors [27].

### 2.4. Handling of Data Imbalance

In this study, there was a sample imbalance between good and nonadherence patients, with a ratio close to 8 : 2. ML classifiers trained on class-imbalanced data are prone to overpredict the majority class. This leads to a larger misclassification rate for the minority class [28]. In this study, the thresholding method was used to reduce the disturbance of imbalance on model training. For binary data, the classification threshold is set to 0.5 by default; however, this is often not ideal for unbalanced data. Adjusting the decision threshold is a good strategy to deal with the class imbalance problem [29, 30]. The decision thresholds of different models were automatically determined according to AUC_ROC. In our study, the optimal decision thresholds for LR, MLP, RF, SVM, and XGBT were 0.498, 1.926*e* − 17, 0.184, 0.756, and 0.323, respectively.

In addition, some of the feature values were unbalanced, such as opioid type, pain causes, and health insurance type. The data were trained and validated by support vector machine (SVM) and logistic regression (LR), and it was found that the performance indicators before and after the removal of such features were almost the same, thus determining that these features did not contribute much. Similarly, after training by the random forest (RF) algorithm, these features were found to be the least important, which affirmed the previous judgment. Therefore, these few metrics are removed in the later model training to increase the model's robustness.

### 2.5. Data Partition

Twenty percent of the samples are randomly selected as the test set, and the remaining 80 percent of the samples are used in the model training process using the k-fold cross-validation method, with *K* taking a value of 10. The cross-validation technique is used to minimize bias in performance comparison and assessment. The testing set was used to evaluate the predictive performance of the five models from the training set.

### 2.6. Model Training and Evaluation

A total of five ML algorithms, including the integrated learning models RF and eXtreme Gradient Boosting (XGBoost), the deep learning model multilayer perceptron (MLP), and the classical binary classification models SVM and LR, were used for model training and prediction of the dataset. Random forest operates by producing a large number of decision trees and is trained by performing bagging operations to combine multiple decision trees or models to arrive at a more stable and accurate data prediction, and is mainly used for classification and regression [31]. XGBoost is based on a sparsity-aware algorithm, a weighted quantile sketch in which weak learners can converge sequentially to the ensemble to achieve a strong learner [32]. MLP is based on feed-forward artificial neural network models, which is important in nonlinear fitting analysis due to its high fault tolerance and adaptivity [33]. SVM is an optimal classification algorithm that distinguishes between different classes of samples in a high-dimensional space and is able to transform the training data into a high-dimensional feature space and obtain a linear optimal solution by separating the hyperplanes with the smallest distances between hyperplane points and the largest margin between classes [34]. LR is a widely used classification model that fits data into a logistic function, enabling prediction of the probability of event occurrence [35]. In each model, the GridSearchCV algorithm was used for hyperparameter tuning with AUC_ROC as the evaluation indicator. Supplementary Table 1 lists the set of hyperparameters and the parameters of the five models.

The five models were evaluated by area under the receiver operating characteristic curve (AUC_ROC), accuracy, specificity, sensitivity, precision, and F1 score. However, model performance is especially influenced by data class proportions and predicted class proportions. As the classes become more imbalanced, it is often the case that we can get good accuracy by simply predicting the majority class, and accuracy cannot be correct for random chance levels that vary due to predicted and data class proportions. Thus, in this study, Cohen's kappa was used to test the statistical significance of model performance against the chance level [36]. The value of the best model for clinical application was assessed using decision curve analysis (DCA).

### 2.7. Additional Statistics

If not stated differently, categorical variables are reported as count (%), and continuous variables are reported as Mean ± SD when they obey a normal distribution; otherwise, they are expressed as median (interquartile range, IQR). A two-sided level of significance of 0.05 was applied to general comparisons. *T*-test, Mann–Whitney test, Chi-square test, and Cohen's kappa statistic were performed using IBM SPSS statistics 27.0 software and R software (version 4.3). All five ML models were operated using Python sklearn.

## 3. Results

### 3.1. Data Collected

A total of 1195 patients were included in the final analysis, of which 945 had good adherence and 250 had poor adherence. The mean age of the participants was 56.41 ± 11.46 years, 61.7% were male patients, the time since onset of pain symptoms was 3.01 ± 4.50 months, the mean number of analgesics per person was 1.32 ± 0.58, the ATC opioid was oxycodone extended-release tablets in 69.94% of the patients, the mean oral oxycodone equivalent daily dose (OEDD) was 37.61 ± 32.89 mg, and 65.36% of patients had good pain control. The demographic and clinical characteristics of the patient are outlined in Tables 1 and 2. Univariate analysis showed that 11 factors were associated with opioid adherence. Patients who were adherent to opioids more frequently had higher family income, shorter time to diagnose, shorter time since onset of pain symptoms, using fentanyl transdermal patch, a higher percentage of nonmixed pain, a higher percentage of pain due to treatment, better pain control, higher BMQ-necessity scores, and lower BMQ-concerns, BMQ-harm, and BMQ-overuse scores compared to that nonadherence.

### 3.2. Model Performance Evaluation

In this study, five selected models, namely, RF, XGBoost, MLP, SVM, and LR, were trained and validated via 10-fold cross-validation. The effectiveness of ML models was assessed by accuracy evaluation and consistency evaluation, including ROC_AUC, accuracy, precision, sensitivity, specificity, F1 score, and Cohen's kappa coefficient.

**Figure 2:**
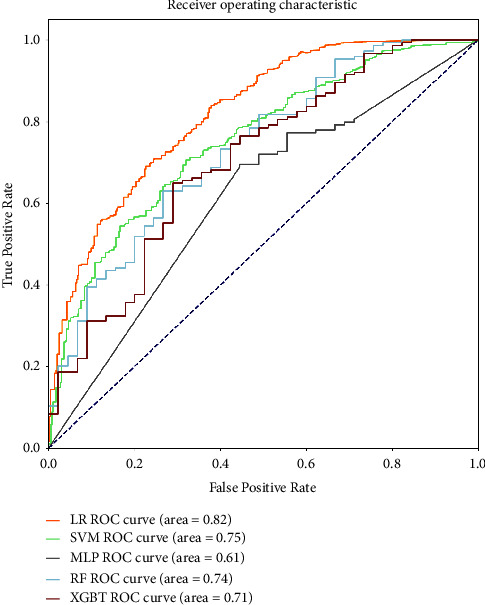
ROC curves with mean AUCs from cross-validation of the five different models on test set data. ROC, receiver operating characteristic; AUC, area under curve; RF, random forest; XGBoost, eXtreme gradient boosting; MLP, multilayer perceptron; SVM, support vector machine, LR, logistic regression.

Figure 2 summarizes the prediction performance on test set data for all the models developed. The AUCs of the four models, LR, SVM, RF, and XGBT, were higher than 0.70, indicating good performance in this classification task, especially the LR model, which had an AUC of 0.82, accuracy of 0.82, and the highest F1 score of 0.88. At the same time, Cohen's kappa of the LR model was 0.22 (*p* < 0.001), which indicated that this model got an accuracy above a certain chance level. The F1 score integrates the model's ability to distinguish negative samples and identify positive samples, and the higher the F1 score, the more robust the model is. Of note, the goal is to predict patients who are less likely to adhere to their treatment plan so that further interventions can be made with this group of patients before taking the medication to improve adherence rates. That is, the prediction of these unpredictable individuals is more important than those adherers. Therefore, the “specificity” describing the “true negative rate” was the focus of this study. Recognizing this, we can see from Table 3 that the LR model had the highest specificity value of 0.71 among all models. Therefore, LR is the best model by combining the results of all aspects.

**Figure 3:**
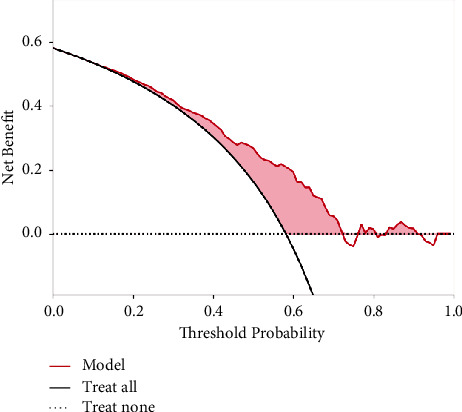
DCA of the LR model on the test set data. The *X*-axis is the threshold probability, and the *Y*-axis is the net benefit rate. The red line indicates the net benefit received by patients when clinical interventions are performed according to the LR model, calculated as true negative diagnosed by the model × the value of benefit from applying clinical interventions to true negative patients − false negative diagnosed by the model × the value of loss from applying clinical interventions to false-negative patients. The curve “Treat all” indicates the benefit of treating all patients as opioid nonadherent and administering the clinical intervention, and the line “Treat none” indicates the benefit of treating all patients as well as adherent to opioids and not giving the intervention. As shown, LR has a greater benefit than all patients receiving or none of the clinical interventions for most of the threshold probability range. LR, logistic regression; DCA, decision curve analysis.

The DCA of test data demonstrated that the LR model showed a higher net benefit than all patients receiving the intervention or no intervention over most of the threshold probability range (Figure 3). Clinical interventions for patients at high risk of opioid nonadherence based on the LR model can benefit patients.

### 3.3. Feature Importance

**Figure 4:**
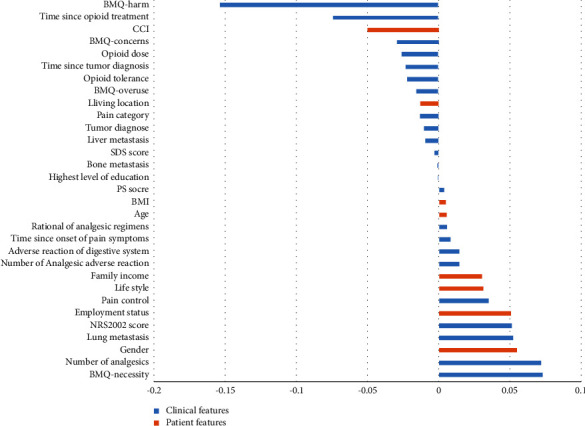
Importance of predictor variables for nonadherence in logistic regression model. BMQ, beliefs about medicines questionnaire; CCI, Charlson comorbidity index; SDS, self-rating depression scale; PS, performance status; BMI, body mass index; NRS2002, nutritional risk screening 2002. When the coefficient is less than zero, it indicates that the greater the value taken for the characteristic, the greater the likelihood that the patient will experience nonadherence, and vice versa. The larger the absolute value of the coefficient, the greater the contribution of the eigenvalue to the prediction of adherence.

To investigate the contribution of each predictive feature in the LR model, we retrieved the coefficients of the elements in the model decision function from each cross-validation fold to serve as the importance of the feature values and summarized the results as shown in Figure 4. When the coefficient is less than zero, it indicates that the greater the value taken for the characteristic, the greater the likelihood that the patient will experience nonadherence, and vice versa. The larger the absolute value of the coefficient, the greater the contribution of the eigenvalue to the prediction of adherence. It can be seen that BMQ-harm, time since the start of opioids, BMQ-necessity, number of analgesics, and gender were the top five features contributing to model predictions. Overall, patient features contributed 25.30%, while clinical features contributed 74.70% toward model predictions (Figure 4).

## 4. Discussion

In this secondary analysis of an exploratory study of genetic and clinical factors associated with opioid response in Chinese Han Chinese patients with cancer pain [25], we constructed five ML models to predict opioid nonadherence in cancer pain patients. By comparing the specificity, sensitivity, accuracy, precision, AUC_ROC, and F1 score of different prediction models, it was concluded that LR was a desirable prediction model, capable of making more accurate predictions, and its specificity for predicting opioid nonadherence in cancer pain patients reached 0.71; i.e., the proportion of truly nonadherent patients was 71% among all patients who were predicted to be nonadherent. While the MLP model performed the worst, DCA shows that the LR model based on demographic and clinical characteristics has high clinical application value in predicting the adherence to opioids in cancer pain patients and is of great clinical significance in guiding healthcare providers on whether early intervention is needed.

Although studies related to predictive models of medication adherence were found in patients with tuberculosis [37], heart failure [38], diabetes [14], self-inject medication at home [24], nicotine withdrawal treatment [23], opioid use disorder treatment [39], and initiation of statin therapy [40], the models constructed in these studies had AUCs of 0.55–0.87, accuracy of 76%–82%, and specificity of 53.6%–78.3%. However, there are no predictive models for opioid adherence in patients with cancer pain, for whom medication adherence is a key factor in treatment outcomes. The best model obtained in this study, the LR model, had an AUC of 0.82, accuracy of 0.82, and specificity of 0.71, indicating a good predictive performance compared to the above studies.

There are various ML algorithms (e.g., LR, classification and regression trees, learning vector quantization, SVM, boosting, and deep neural networks) that are used to develop various clinical event prediction models. LR is a traditional binary classification model in ML, and the simplicity and effectiveness of this algorithm have made it widely used in many fields. Logistic regression prediction models were used in 53% of studies of clinical support decision systems in emergency departments [41]. Christodoulou et al. included 71 studies comparing the ability of LR and other ML models in predicting dichotomous clinical outcomes such as disease diagnosis or prognosis and showed that the performance was close [42]. In the low-risk-of-bias studies, there was no difference on average between the performance of LR models and ML models in terms of AUC, with a mean logit (AUC) difference of 0.00 (−0.18 to 0.18).

Similar to diagnostic metrics, ML prediction models have sensitivity and specificity. When building predictive models, seeking to increase sensitivity will necessarily reduce the specificity of the model predictions [43]. In clinical practice, our goal is to predict patients who are less likely to adhere to their treatment plan, so that further interventions can be introduced to this group of patients prior to planned medication use to improve adherence rates. Therefore, the “specificity,” which describes the “true negative rate,” was the focus of this study. Although 43 demographic and clinical characteristics that may be associated with opioid adherence were included in this study, the specificity of the best model prediction did not reach 0.8. This may be because this study was a secondary analysis and the data used to train the model may not have included other important factors affecting patient adherence, such as caregiver attitudes toward opioids, patient expectations of analgesic treatment, and satisfaction with medical staff. In addition, the patient included in this study had been taking opioids for varying lengths of time, from 72 h to 54 months, which made the prediction of nonadherence more complicated. It is suggested that in the future, prediction models could be developed for different phases of treatment (e.g., initial, implementation, and discontinuation of therapy) to achieve more accurate prediction [44].

Our LR prediction model covered many known risk factors for medication adherence, including BMQ, duration of opioids, number of analgesic medications, gender, and employment status. The BMQ-harm was the most important predictor of nonadherence to opioids in cancer pain patients with an importance coefficient of 0.15. In addition to this, BMQ-concern had an importance coefficient of 0.03. The BMQ-necessary is the most important positive predictor of patient adherence. Over the past decade, many studies have used BMQ to explore the relationship between patients' medication beliefs and adherence [45]. In general, the higher the patient's medication apprehension, the lower the medication adherence [46]. In particular, Chinese patients' long-standing misconceptions about pain and analgesics have led to excessive concerns about analgesic side effects and addiction, and reluctance to use opioids. Even after extensive pain education for patients and their caregivers in recent years, they still maintain very high negative beliefs about pain and analgesic medications [47]. In contrast, BMQ-necessary was positively associated with adherence. Fischer et al. reported a significant positive association between BMQ-necessary and adherence at 3-month follow-up in patients with COPD (OR = 2.6, 95% CI 1.4–5.1) [48]. In addition to the known risk factors, our LR prediction model uncovered some new influences, such as CCI. No literature was retrieved to confirm the relationship between CCI and medication adherence, and the investigators hypothesized that a higher CCI indicates that the patient has more comorbidities or more serious comorbidities and is more likely to have a combination of multiple medications, and poly-medication is one of the risk factors for nonadherence to analgesic medications [49].

The development of an adherence prediction model can help identify patients at high risk of opioid nonadherence promptly and can better inform clinical staff of interventions to improve medication adherence, thereby enabling individualized education and care for patients with cancer pain. In actual clinical practice, clinicians generally do not notice treatment nonadherence until after patients have experienced it multiple times, and then take action. If clinicians were able to predict the risk of opioid nonadherence in advance, this would allow them to proactively adopt intervention strategies and focus their attention and efforts on high-risk patients, reducing unnecessary interventions in those patients with high adherence.

Limitations of this study include (1) the small sample size of the dataset used for our study, the limited number of features, and the high imbalance between adherent and nonadherent samples, resulting in the model constructed that is not yet sufficiently stable and has room for further optimization; (2) only 80% was used as threshold to classify patients as adherent or nonadherent, so the stability and applicability of the model under other thresholds cannot be examined; (3) as this study was a secondary analysis, it lacked necessary information to further classify adherence into different subtypes, and to develop a comprehensive model to predict different types of nonadherence; (4) the monocentric nature of the data is a limitation of the study, and it is unclear whether model performance is extrapolated to other healthcare institutions. In future studies, multicenter standardized data could be assembled for training and validation of the model.

There are numerous previous studies on factors influencing opioid adherence in patients with cancer pain, but few studies on predictive models of opioid adherence in patients with cancer pain. To the best of our knowledge, this study is the first to develop prediction models based on these risk factors using an ML approach. In our study, five ML-based modeling algorithms were developed for adherence prediction, and after comparing the specificity, sensitivity, accuracy, precision, AUC, and F1 score, we concluded that LR was the most optimal model, and showed a good predictive ability to identify nonadherent patients. Future studies can develop more accurate prediction models for cancer pain patients at different treatment stages, include patients from multiple centers and levels of hospitals, increase the representativeness of the sample, and continuously revise and improve the prediction model for opioid adherence in cancer pain patients. In addition, programming the prediction model to design an automated tool for predicting adherence, which may then be used by clinicians, pharmacists, and nurses as a decision support tool could be explored to improve cancer pain management.

In conclusion, this study developed five ML-based modeling algorithms for opioid nonadherence prediction, with LR being the most desirable model and showing good predictive power for identifying nonadherent patients. The model identified several important predictors of opioid nonadherence in patients with cancer pain, and this information may help clinical staff identify patients at high risk of nonadherence and develop targeted intervention strategies to promote patient adherence and improve patient benefit.

## Figures and Tables

**Table 1:** Demographic characters of participants.

Variables	All (*n* = 1195)	Adherers (*n* = 945)	Nonadherers (*n* = 250)	*p* value	Missing
Age (years)	1195				0
Mean ± SD	56.41 ± 11.46	56.28 ± 11.59	56.88 ± 11.01	0.469	
Ethnic group	1195				0
Han (*n* (%))	1187 (99.33)	938 (99.26)	249 (99.60)	0.880	
Others	8 (0.67)	7 (0.74)	1 (0.40)		
Gender	1195			0.500	0
Male	738 (61.76)	579 (61.27)	159 (63.60)		
Female	457 (38.24)	366 (38.73)	91 (36.40)		
BMI (kg/m^2^)	1195			0.809	0
Mean ± SD	21.17 ± 3.25	21.16 ± 3.18	21.22 ± 3.52		
Marital status	1195				0
Married	1167 (97.66)	925 (97.88)	242 (96.80)	0.219	
Divorced/widowed	9 (0.75)	5 (0.53)	4 (1.60)		
Unmarried	19 (1.59)	15 (1.59)	4 (1.60)		
Family income (CNY)	1194			<0.001	0
<1000	91 (7.62)	54 (5.72)	37 (14.80)		
1000∼2000	269 (22.53)	215 (22.78)	54 (21.60)		
2000∼3000	428 (35.82)	345 (36.51)	83 (33.20)		
≥3000	407 (34.09)	331 (35.06)	76 (30.40)		
Highest level of education	1195			0.215	0
Illiteracy	56 (4.69)	39 (4.13)	17 (6.80)		
Primary or junior high school	656 (54.90)	531 (56.19)	125 (50.00)		
Senior school	317 (26.53)	243 (25.71)	74 (29.60)		
College or above	165 (13.81)	131 (13.86)	34 (13.60)		
Health insurance	1195			0.893	0
Yes	1163 (97.32)	920 (97.35)	243 (97.20)		
No	32 (2.68)	25 (2.65)	7 (2.80)		
Living location	1195			0.068	0
Urban	305 (25.52)	230 (24.34)	75 (30.00)		
Town	890 (74.48)	715 (75.66)	175 (70.00)		
Employment status	1195			0.946	0
Employed	560 (46.86)	441 (46.67)	119 (47.60)		
Retried	476 (39.83)	379 (40.11)	97 (38.80)		
Unemployed	152 (12.72)	120 (12.70)	32 (12.80)		
Student	7 (0.59)	5 (0.53)	2 (0.80)		
Life style	1195			0.111	0
Living alone	8 (0.67)	4 (0.42)	4 (1.60)		
Living with family	1187 (99.33)	941 (99.58)	246 (98.40)		

SD, standard deviation; BMI, body mass index; CNY, Chinese Yuan; BMQ, beliefs about medicines questionnaire; mo, month; CCI, Charlson comorbidity index; SDS, self-rating depression scale; PS, performance status; NRS2002, nutritional risk screening 2002.

**Table 2:** Clinical data of participants.

Variables	All (*n* = 1195)	Adherers (*n* = 945)	Nonadherers (*n* = 250)	*p* value	Missing
Tumor diagnosis	1195			0.607	0
Lung	480 (40.17)	373 (39.47)	107 (42.80)		
Gastrointestinal	298 (24.94)	245 (25.93)	53 (21.20)		
Breast	84 (7.03)	63 (6.67)	21 (8.40)		
Genitourinary	77 (6.44)	59 (6.24)	18 (7.20)		
Head and neck	101 (8.45)	81 (8.57)	20 (8.00)		
Lymphohematopoietic	24 (2.01)	20 (2.12)	4 (1.60)		
Unknown origin	18 (1.51)	16 (1.69)	2 (0.80)		
Others	121 (10.13)	92 (9.74)	29 (11.60)		
Time of tumor diagnosis (mo)	1190				5
Median (IQR)	7.00 (1.30, 18.30)	6.20 (1.20, 17.00)	8.55 (2.40, 24.20)	0.005	
Tumor metastasis status	1195			0.414	0
None	137 (11.46)	112 (11.85)	25 (10.00)		
Bone	594 (49.71)	466 (49.31)	128 (51.20)		
Liver	166 (13.89)	137 (14.50)	29 (11.60)		
CNS	312 (26.11)	240 (25.40)	72 (28.80)		
Lung	238 (19.92)	194 (20.53)	44 (17.60)		
PS	1195			0.371	0
0-1	921 (77.07)	661 (69.95)	183 (73.20)		
>1	274 (22.93)	284 (30.05)	67 (26.80)		
NRS2002 score	1195			0.081	0
Median (IQR)	3 (2.4)	3 (2.4)	3 (2.4)		
SDS score	1193				2
Mean ± SD	42.89 ± 9.47	42.97 ± 9.44	42.78 ± 9.22	0.808	
Charlson comorbidity index (CCI)	1195			0.081	0
<7	347 (29.04)	283 (29.95)	64 (25.60)		
≥7	848 (70.96)	662 (70.05)	186 (74.40)		
Opioid tolerance	1195			0.763	0
No	886 (74.14)	703 (74.39)	183 (73.20)		
Yes	309 (25.86)	242 (25.61)	67 (26.80)		
Time since onset of pain symptoms (mo)	1174				
Median (IQR)	1.50 (1.00, 3.00)	1.50 (1.00, 3.00)	2.00 (1.00, 4.00)	0.006	21
Time since opioid treatment (mo)	1195				0
Median (IQR)	0.33 (0.1∼1.0)	0.30 (0.1∼1.0)	0.50 (0.10, 1.50)	0.098	
Pain category	1195			0.004	0
Bone soft-tissue pain	845 (70.71)	687 (72.70)	158 (63.20)		
Visceral pain	132 (11.05)	96 (10.16)	36 (14.40)		
Neuropathic pain	17 (1.42)	14 (1.48)	3 (1.20)		
Mixed pain	201 (16.82)	148 (15.66)	53 (21.20)		
Causes of pain	1195			0.049	0
Tumor pressing	1138 (95.23)	849 (89.84)	244 (97.60)		
Cancer treatment	56 (4.69)	50 (5.29)	6 (2.40)		
Other diseases	2 (0.17)	1 (0.11)	1 (0.40)		
Number of analgesics	1195				0
Mean ± SD	1.32 ± 0.58	1.34 ± 0.59	1.28 ± 0.54	0.357	
Opioid around-the-clock	1195			0.004	0
Oxycodone hydrochloride prolonged-release tablets	836 (69.96)	652 (68.99)	184 (73.60)		
Morphine sulfate sustained-release tablets	16 (1.34)	10 (1.06)	6 (2.40)		
Fentanyl transdermal patch	203 (16.99)	178 (18.84)	25 (10.00)		
Oxycodone and paracetamol tablet	55 (4.60)	39 (4.13)	16 (6.40)		
Weak opioids	9 (0.75)	6 (0.63)	3 (1.20)		
Combined use of two or more long-acting preparations	76 (6.36)	60 (6.35)	16 (6.40)		
Opioid dose(OEDD, mg)	1195			0.426	0
Mean ± SD	37.61 ± 32.89	37.77 ± 33.61	37.01 ± 30.08		
Median (IQR)	25 (20, 40)	25 (20, 40)	25 (20, 40)		
Rational of analgesic regimens	1195			0.172	0
No	245 (20.50)	186 (19.68)	59 (23.60)		
Yes	950 (79.50)	759 (80.32)	191 (76.40)		
Pain control	1195			<0.001	0
Poor	414 (34.64)	303 (32.06)	111 (44.40)		
Good	781 (65.36)	642 (67.94)	139 (55.60)		
BMQ					
Specific-necessity	1055				140
Mean ± SD	19.13 ± 2.92	19.40 ± 2.74	18.07 ± 3.35	<0.001	
Specific-concerns	1055				140
Mean ± SD	15.46 ± 4.61	14.92 ± 4.59	17.54 ± 4.05	<0.001	
General-harm	1035				160
Mean ± SD	15.46 ± 4.61	12.65 ± 3.55	15.88 ± 3.65	<0.001	
General-overuse	1035				160
Mean ± SD	6.18 ± 2.20	5.98 ± 2.03	6.95 ± 2.62	<0.001	
Number of analgesic adverse reaction	1195				0
Mean ± SD	0.92 ± 0.86	0.93 ± 0.86	0.88 ± 0.84	0.894	
Systems involved with ADR	1195				0
Central nervous system	141 (11.8)	119 (12.59)	22 (8.80)	0.098	
Digestive system	755 (63.18)	596 (63.07)	159 (63.60)	0.877	
Skin	14 (1.17)	11 (1.16)	3 (1.20)	0.963	
Others	124 (10.38)	105 (11.11)	18 (7.20)	0.070	

SD, standard deviation; OEDD, oral oxycodone equivalent daily dose; IQR, interquartile range; BMQ, beliefs about medicines questionnaire; mo, month; CCI, Charlson comorbidity index; SDS, self-rating depression scale; PS, performance status; NRS2002, nutritional risk screening 2002.

**Table 3:** Comparisons of different models in terms of five performance metrics: accuracy, precision, sensitivity, specificity, F1 score, and Cohen's kappa.

Models	Accuracy	Precision	Sensitivity	Specificity	F1 score	Kappa	*p* value
RF	0.79	0.88	0.87	0.37	0.87	0.22	<0.001
XGBT	0.74	0.89	0.79	0.45	0.83	0.19	<0.001
LR	0.82	0.90	0.85	0.71	0.88	0.53	<0.001
MLP	0.57	0.87	0.57	0.55	0.69	0.07	0.172
SVM	0.80	0.88	0.89	0.32	0.88	0.21	<0.001

When 0< kappa <1, it means that classifiers get an accuracy above chance level. RF, random forest; XGBoost, eXtreme Gradient Boosting; MLP, multilayer perceptron; SVM, support vector machine, LR, logistic regression.

## Data Availability

All relevant data are included within this article. Any additional information is available from the corresponding author upon reasonable request.

## Supplementary Materials

Supplementary Materials The set of hyperparameters and the parameters in five ML models.
