# Supplementary material for: Opioid Nonadherence Risk Prediction of Patients with Cancer-Related Pain Based on Five Machine Learning Algorithms
Source: Pain Res Manag. 2024 Jun 6;2024:7347876. doi: 10.1155/2024/7347876 (PMC11175844; doi:10.1155/2024/7347876)
Supplement: Supplementary Materials — The set of hyperparameters and the parameters in five ML models. [file 7347876.f1.docx]

| Supplementary Table 1. The set of hyperparameters and the parameters in five ML models | |
| --- | --- |
| **Model** | **Hyperparameters** |
| LR | solver: liblinear, random_state: 2, max_iter: 50, penalty: l1, class_weight: {0: 0.64, 1: 0.36} |
| MLP | activation=relu,alpha=1e-05, batch_size=auto, beta_1=0.9, beta_2=0.999, early_stopping=False, epsilon=1e-08, hidden_layer_sizes=(25,25,15,5), learning_rate=constant, learning_rate_init=0.001, max_iter=15000, momentum=0.9, nesterovs_momentum=True, power_t=0.5, random_state=1, shuffle=True, solver=lbfgs, tol=0.0001, validation_fraction=0.1, verbose=False, warm_start=False |
| RF | min_samples_leaf = 2, max_depth = 7, n_estimators = 1200 |
| SVM | gamma: 0.003, C: 10, kernel: linear, degree: 1 |
| XGBT | max_depth=10, learning_rate=0.005, n_estimators=300, silent=False, objective=multi:softprob, booster=gbtree, num_class=2, n_jobs=1, gamma=0.5, min_child_weight=1, subsample=0.8, colsample_bytree=0.7, seed=3 |

ML, machine learning; RF, random forest; XGBOOST, eXtreme Gradient Boosting; MLP, multilayer perceptron; SVM, support vector machine, LR, logistic regression.
